# Supplementary material for: Nanolipid-Trehalose Conjugates and Nano-Assemblies as Putative Autophagy Inducers
Source: Pharmaceutics. 2019 Aug 20;11(8):422. doi: 10.3390/pharmaceutics11080422 (PMC6723367; doi:10.3390/pharmaceutics11080422)
Supplement: Supplementary file 1 [file pharmaceutics-11-00422-s001.pdf]

pharmaceutics

Supplementary Materials: Nanolipid-Trehalose Conjugates and Nano-Assemblies as Putative Autophagy Inducers

Eleonora Colombo, Michele Biocotino, Giulia Frapporti, Pietro Randazzo, Michael S. Christodoulou, Giovanni Piccoli, Laura Polito, Pierfausto Seneci and Daniele Passarella

NA characterization — TEM. NAs were characterized by transmission electron microscopy (Zeiss LIBRA 200FE, equipped with: 200 kV FEG, in column second-generation omega filter for energy selective spectroscopy (EELS) and imaging (ESI), HAADF STEM facility, EDS probe for chemical analysis, integrated tomographic HW and SW system). The samples for TEM analysis were prepared depositing a drop of NA solution (at a concentration of 2 mg/mL) on a carbon-coated copper grid (300 mesh) and evaporating the solvent. The particle size distribution was obtained by measuring at least 100 nanoparticles.

NA characterization — UV. NAs were characterized using an Evolution 600 UV-Vis Thermo Scientific instrument. A disposable cuvette with 1 cm optical path length was used for the out-line measurements. An optical window with a path length of 1 mm was used.

1) **mono-Sq-NA1**

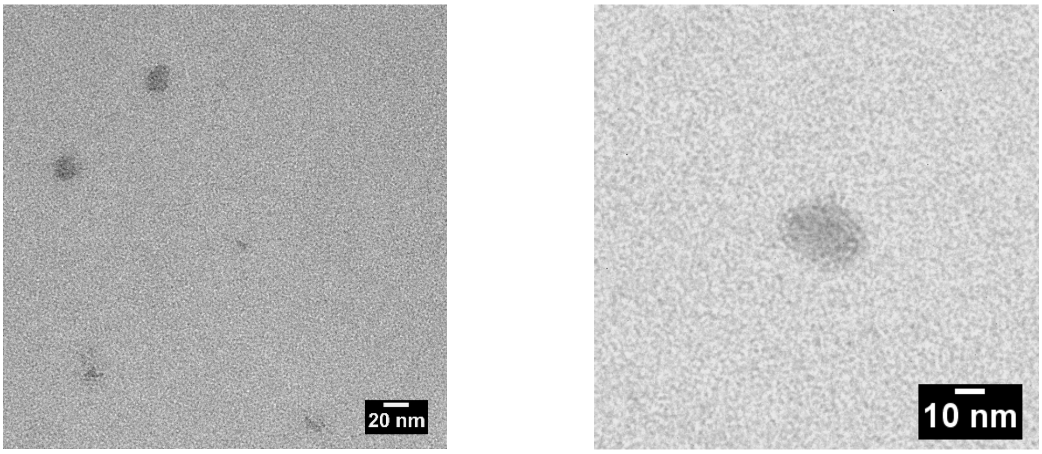

Figure S1. TEM images of **mono-Sq-NA1**. D_{median} = 37.5 ± 7.2 nm.

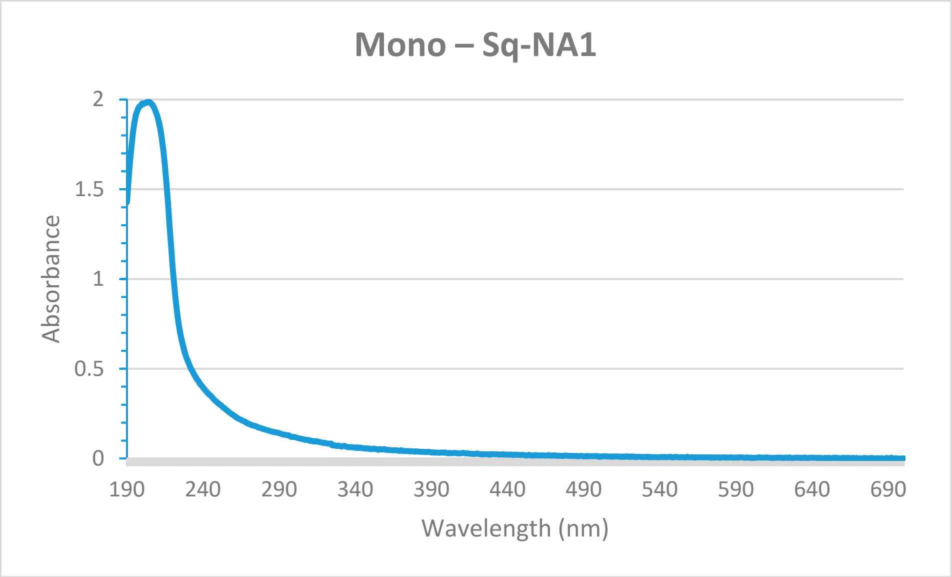

Figure S2. UV-vis spectrum of **mono-Sq-NA1**.

2) bis-Sq-NA2

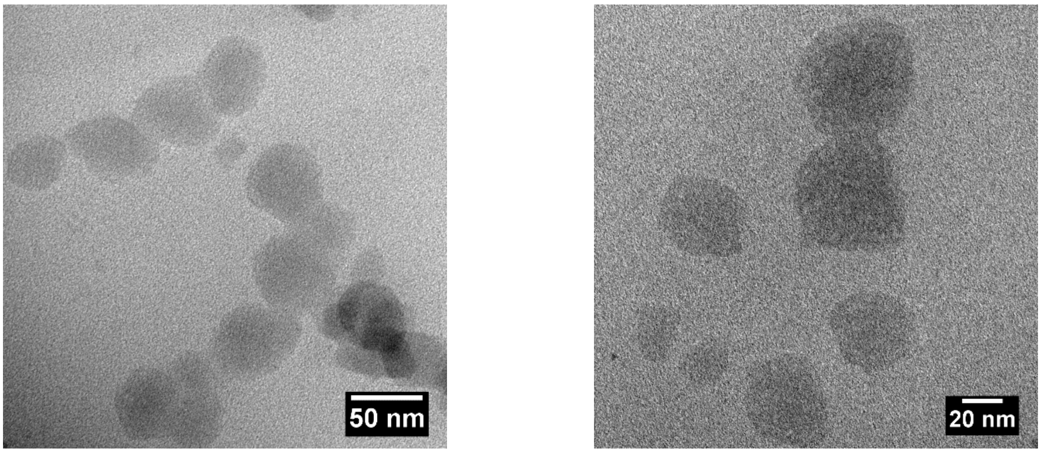

Figure S3. TEM images of **bis-Sq-NA2**. D_{median} = 48.5 ± 11.4 nm.

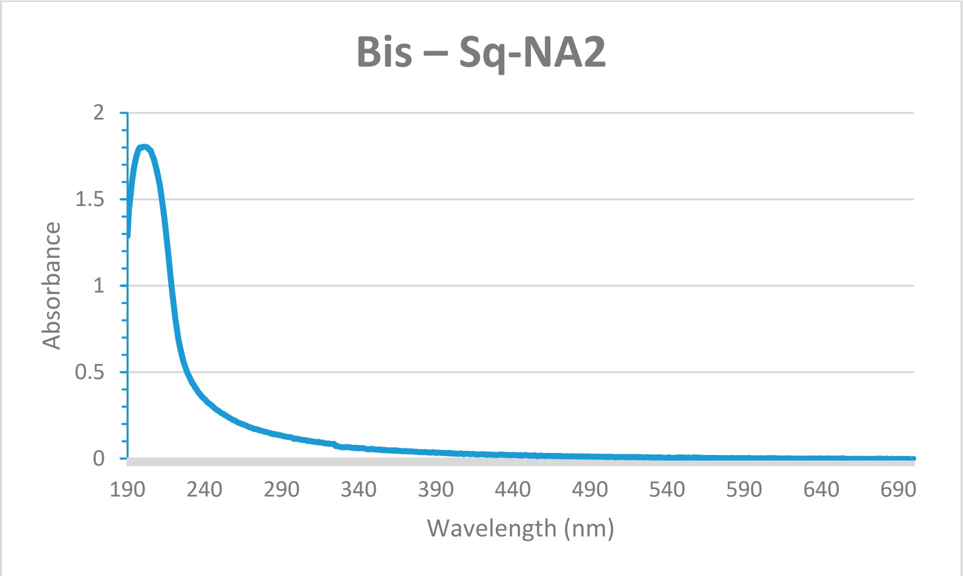

Figure S4. UV-vis spectrum of **bis-Sq-NA2**.

3) mono-Be-NA3

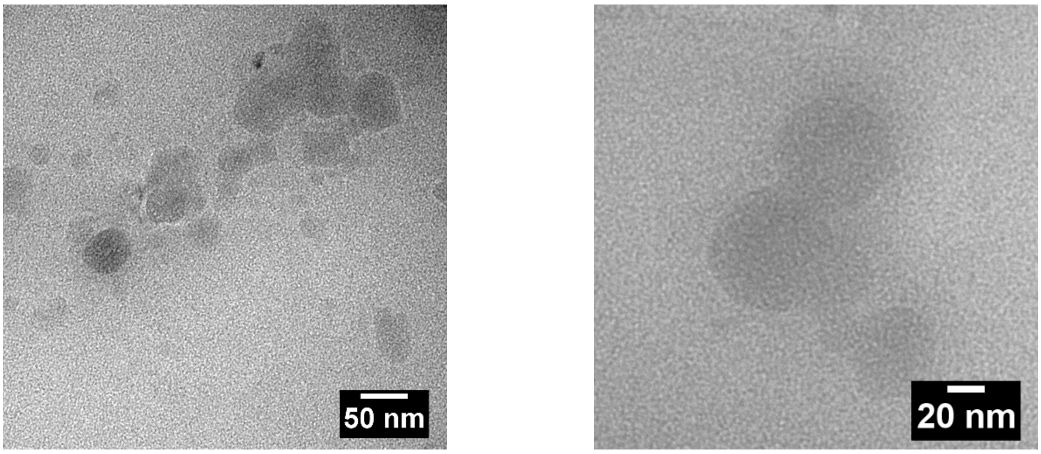

Figure S5. TEM images of **mono-Be-NA3**. D_{median} = 71.1 ± 13.6 nm.

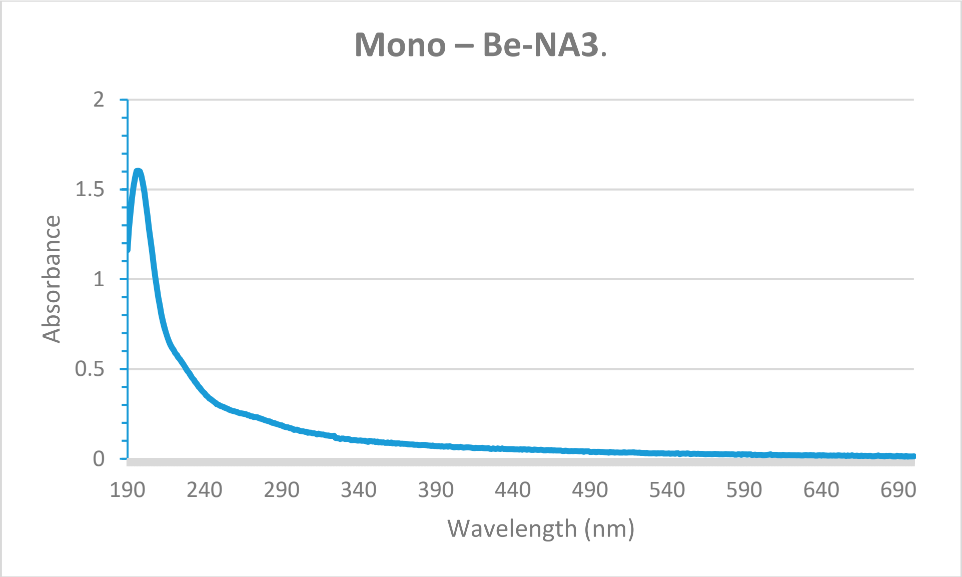

Figure S6. UV-vis spectrum of **mono-Be-NA3**.

Bioanalytical determination of nanolipid-trehalose conjugates, and of their degradation products in cell lysates. HeLa cell lysates from previously described autophagy assays (40 µM concentrations, **mono-Sq-NA1/EC-37** and **mono-Be-NA3/MIC-17**), after centrifugation for 20 min at 16,000g, were prepared for bioanalytical testing [44]. Briefly, MeOH (4 volumes) and chloroform (1 volume) were added, and vortexing was done after each addition. The resulting mixture was centrifuged (15 min at 10,000g), and the surnatant (aqueous methanolic phase for testing) was removed. Then, MeOH (4 volumes) was further added to the mixture, followed by vortexing and centrifugation (15 min at 10,000g); the surnatant (methanolic chloroform organic phase for testing) was also collected.

Each phase was then submitted to UPLC-MS characterization, using an UPLC Acquity Waters System instrument equipped with a single quadrupole detector (SQD) and a tunable UV detector (TUV).

As to LC analysis, an Acquity UPLC BEH SHIELD RP_{18} column, 2.1x50 mm, id = 1.7 µm; a phase A: 0.05% TFA in HiPerSolv water; a phase B: 0.05% TFA in HiPerSolv acetonitrile; a gradient: 3 min run, from 40% to 100% B in 2.8 min, 100% B for 0.2 min; a 0.5 mL/min flux, and a λ = 220 nm were used. As to MS analysis, it was run with ESI^+ ionization mode, with MS-SCAN: 100-2000 uma; and with SCAN time: 0.2 sec.

1) **mono-Sq-NA1/EC-37**

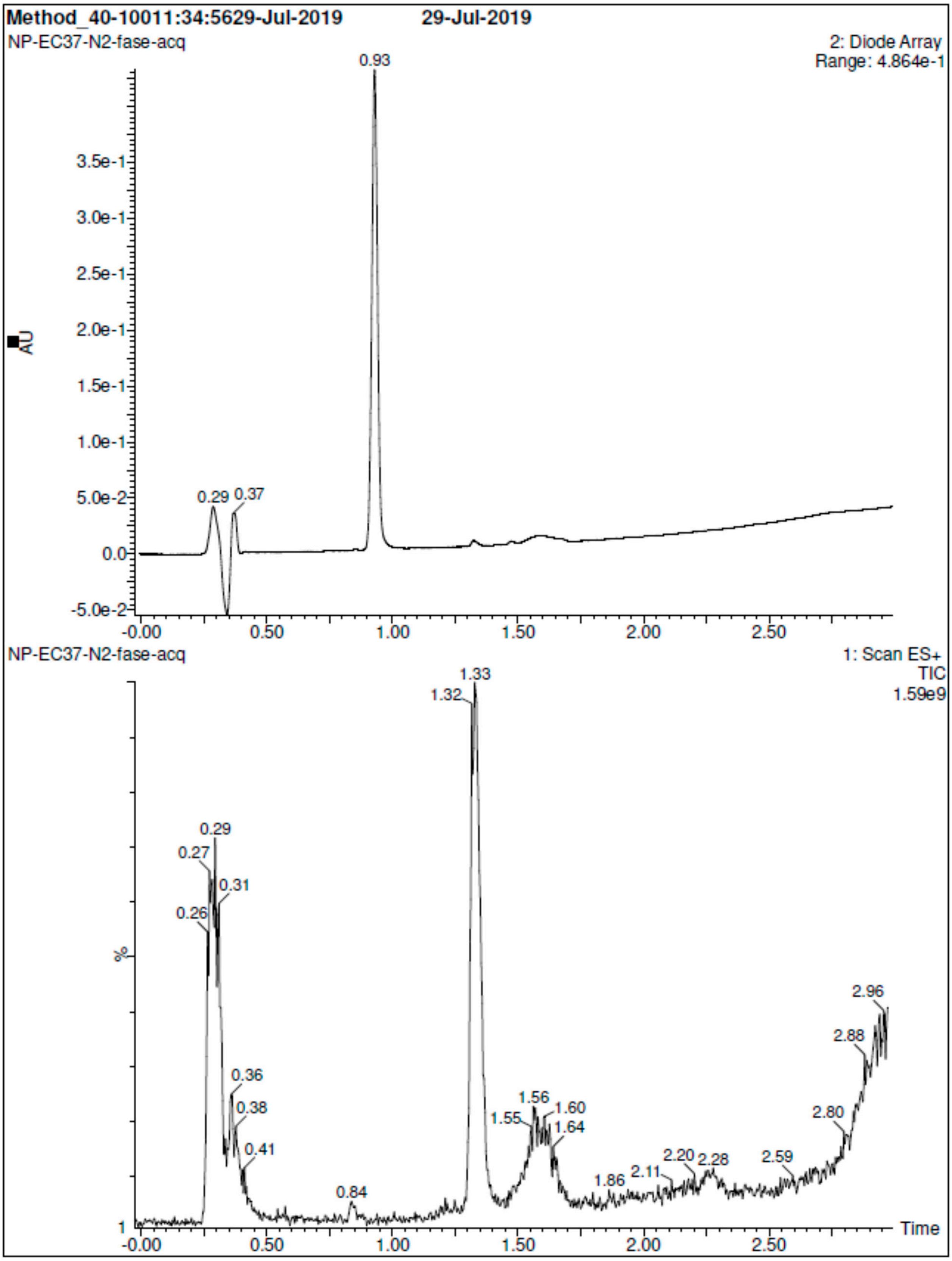

Figure S7. HPLC (220 nm, top) and TIC spectrum (ESI+, bottom) of the aqueous methanolic phase from the treatment of HeLa cells with **mono-Sq-NA1/EC-37**.

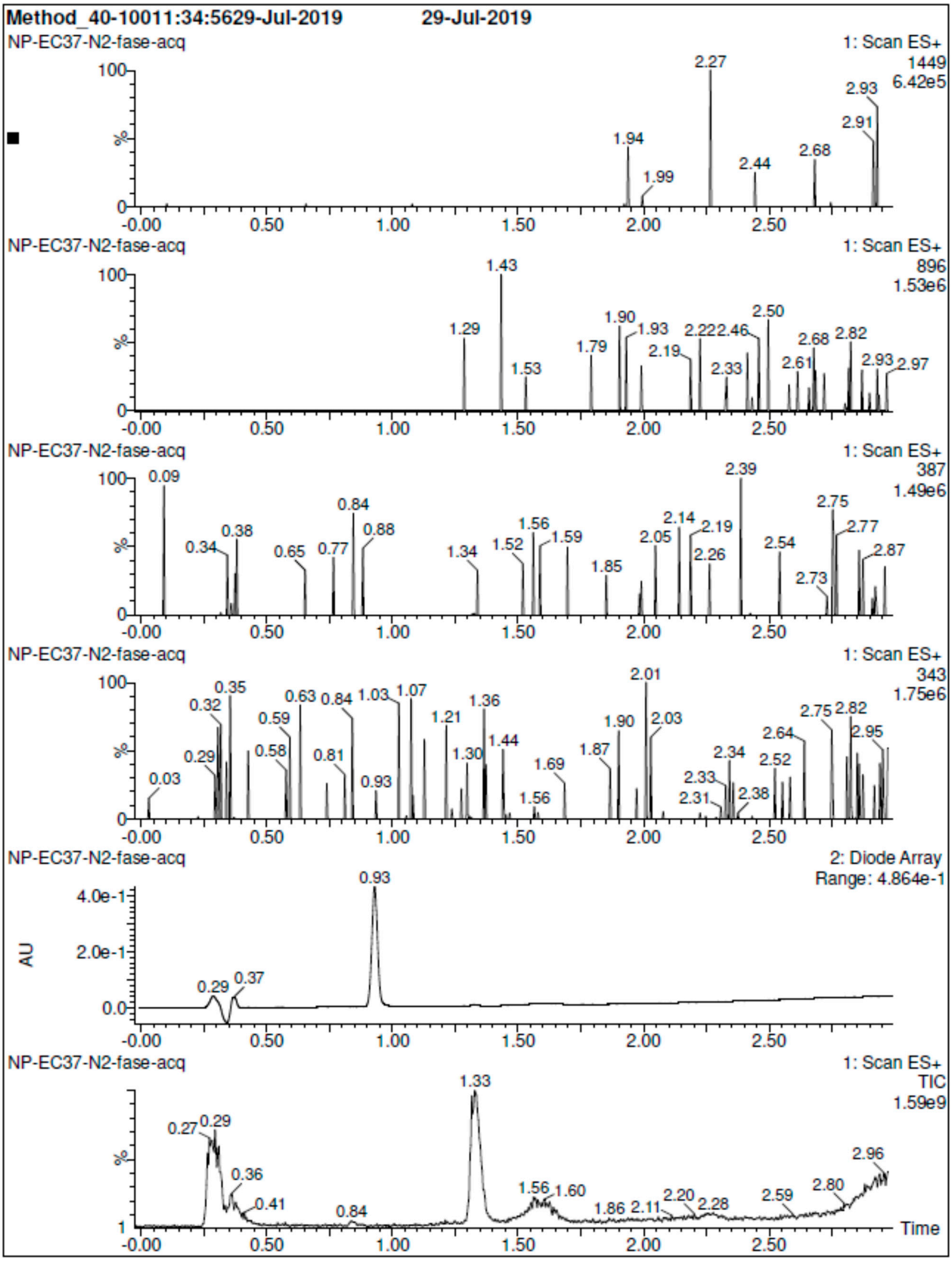

Figure S8. MS analysis / compound searching for **2-Sq-bis** (MW=1448, first lane), **1-Sq-mono** (MW = 895, second lane), squalene alcohol (MW=386, third lane) and trehalose (MW = 342 Da, fourth lane) in the aqueous methanolic phase from the treatment of HeLa cells with **mono-Sq-NA1/EC-37**.

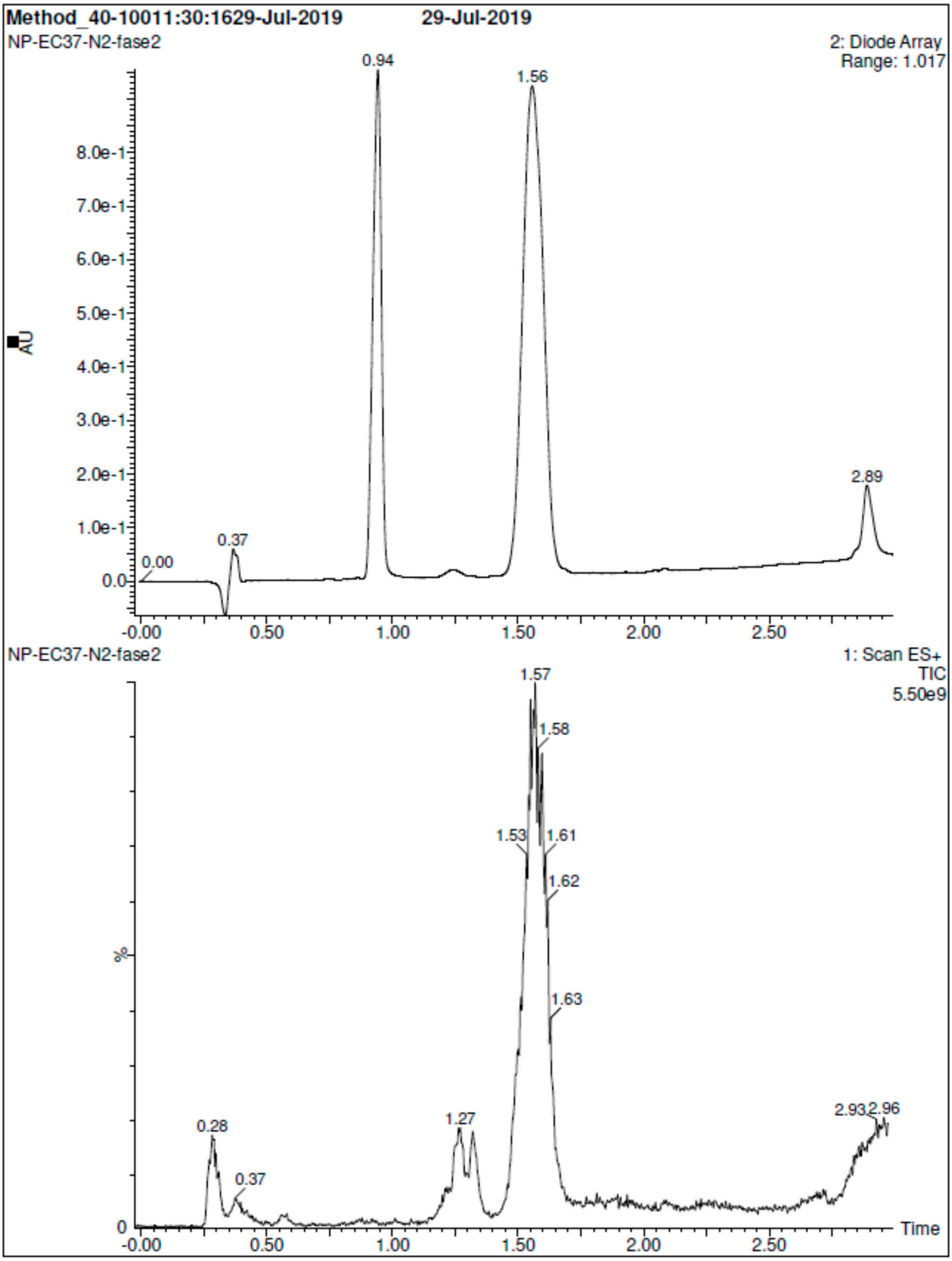

Figure S9. HPLC (220 nm, top) and TIC spectrum (ESI$^+$, bottom) of the lipophilic methanol-chloroform phase from the treatment of HeLa cells with **mono-Sq-NA1/EC-37**.

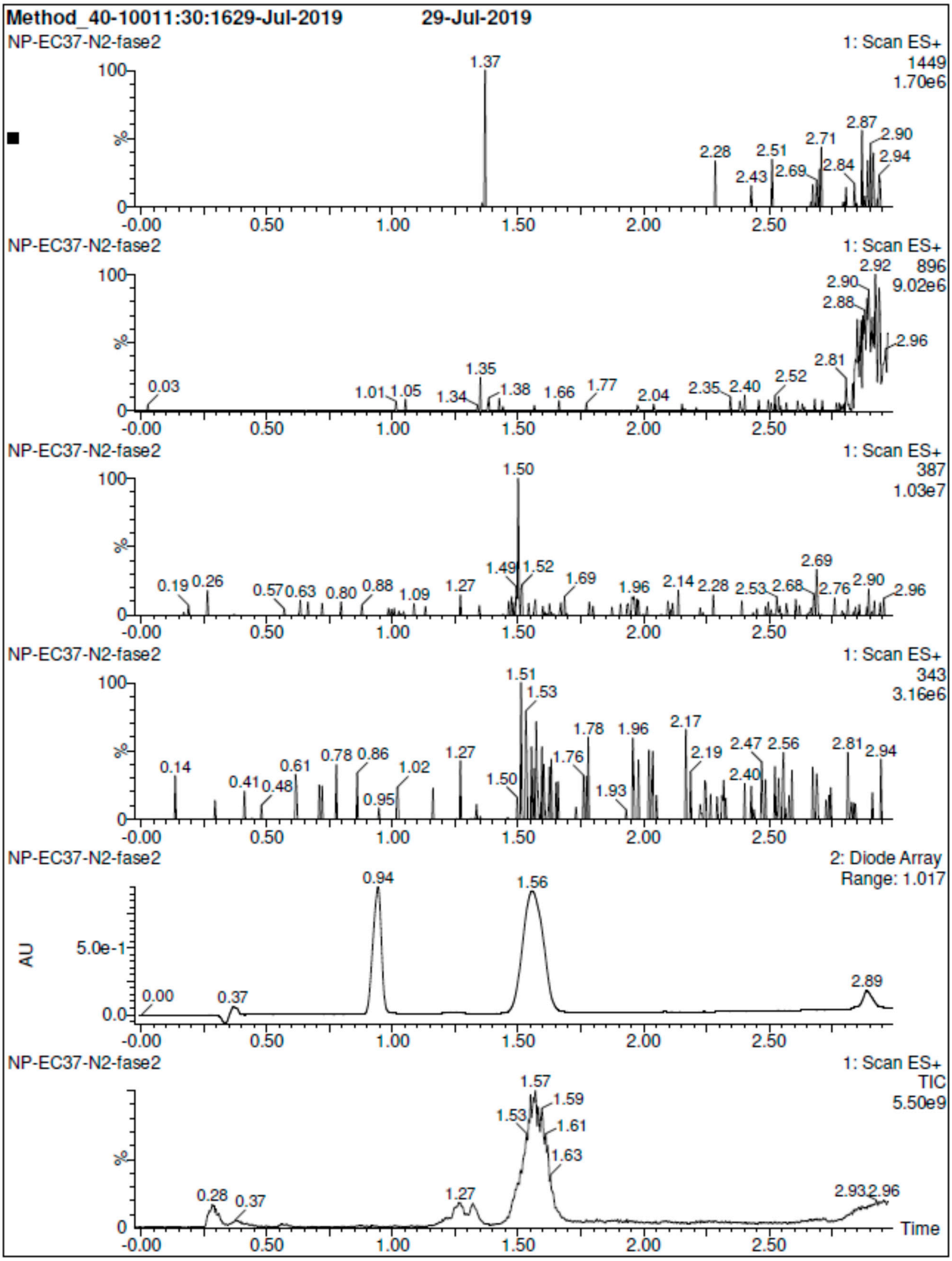

Figure S10. MS analysis / compound searching for **2-Sq-bis** (MW = 1448, first lane), **1-Sq-mono** (MW = 895, second lane), squalene alcohol (MW = 386, third lane) and trehalose (MW = 342 Da, fourth lane) in the lipophilic methanol-chloroform phase from the treatment of HeLa cells with **mono-Sq-NA1/EC-37**.

2) **mono-Be-NA3/MIC-17**

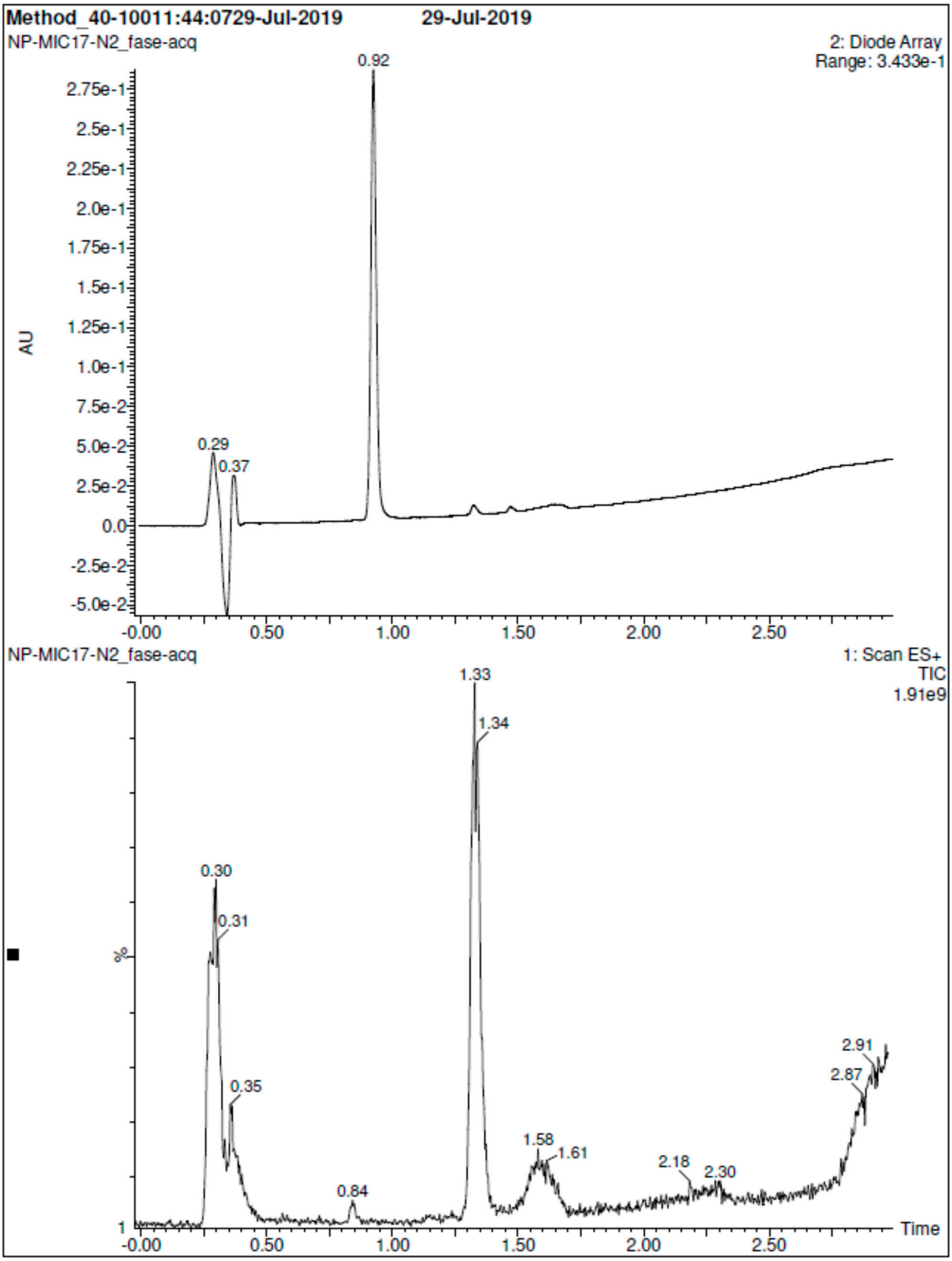

Figure S11. HPLC (220 nm, top) and TIC spectrum (ESI$^+$, bottom) of the aqueous methanolic phase from the treatment of HeLa cells with **mono-Be-NA3/MIC-17**.

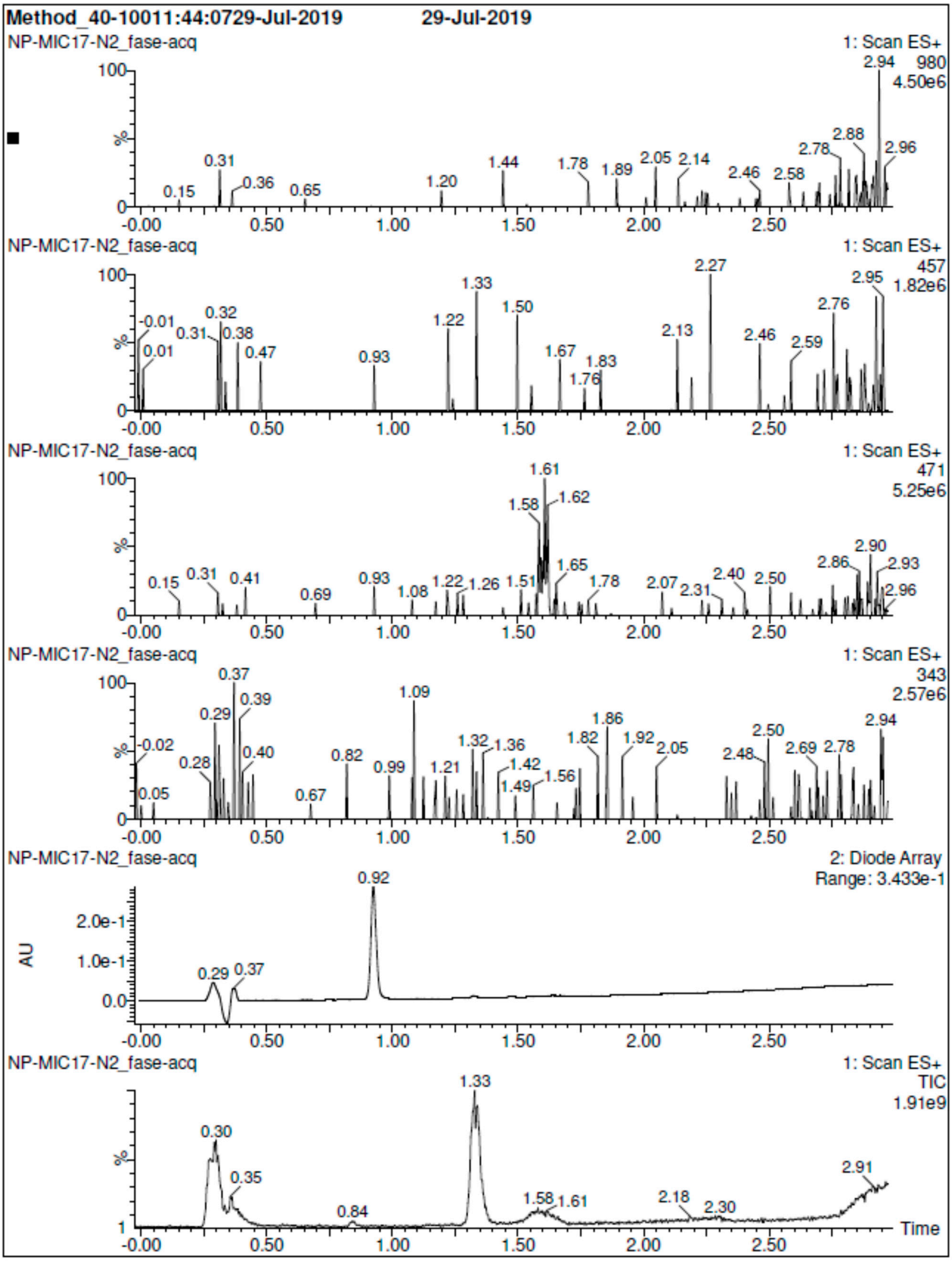

Figure S12. MS analysis / compound searching for **3-Be-mono** (MW = 979, first lane), betulinic acid (MW = 456, second lane), betulinic acid methyl ester (MW = 470, third lane) and trehalose (MW = 342 Da, fourth lane) in the aqueous methanolic phase from the treatment of HeLa cells with **mono-Be-NA3/MIC-17**.

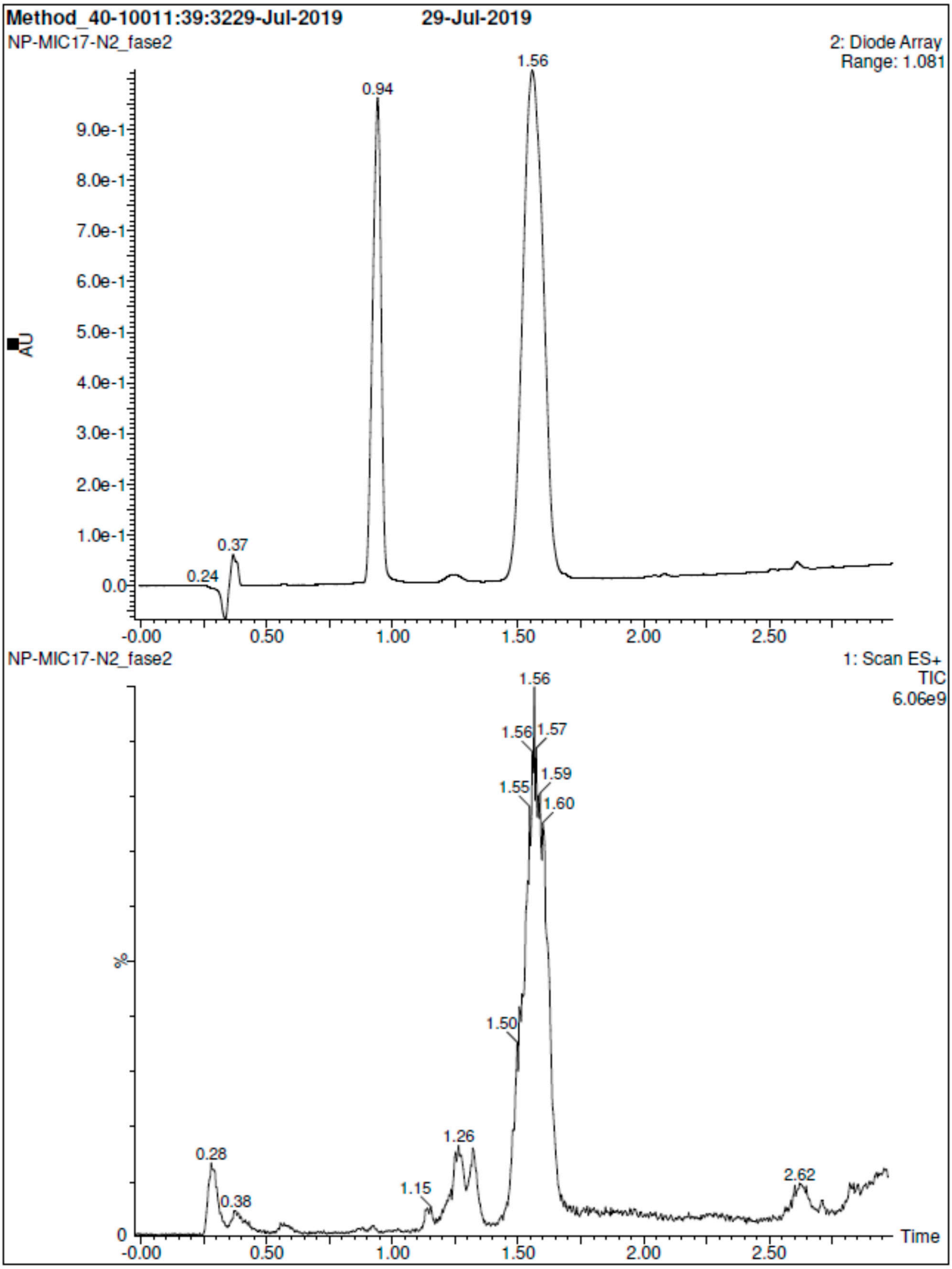

Figure S13. HPLC (220 nm, top) and TIC spectrum (ESI^+, bottom) of the lipophilic methanol-chloroform phase from the treatment of HeLa cells with **mono-Be-NA3/MIC-17**.

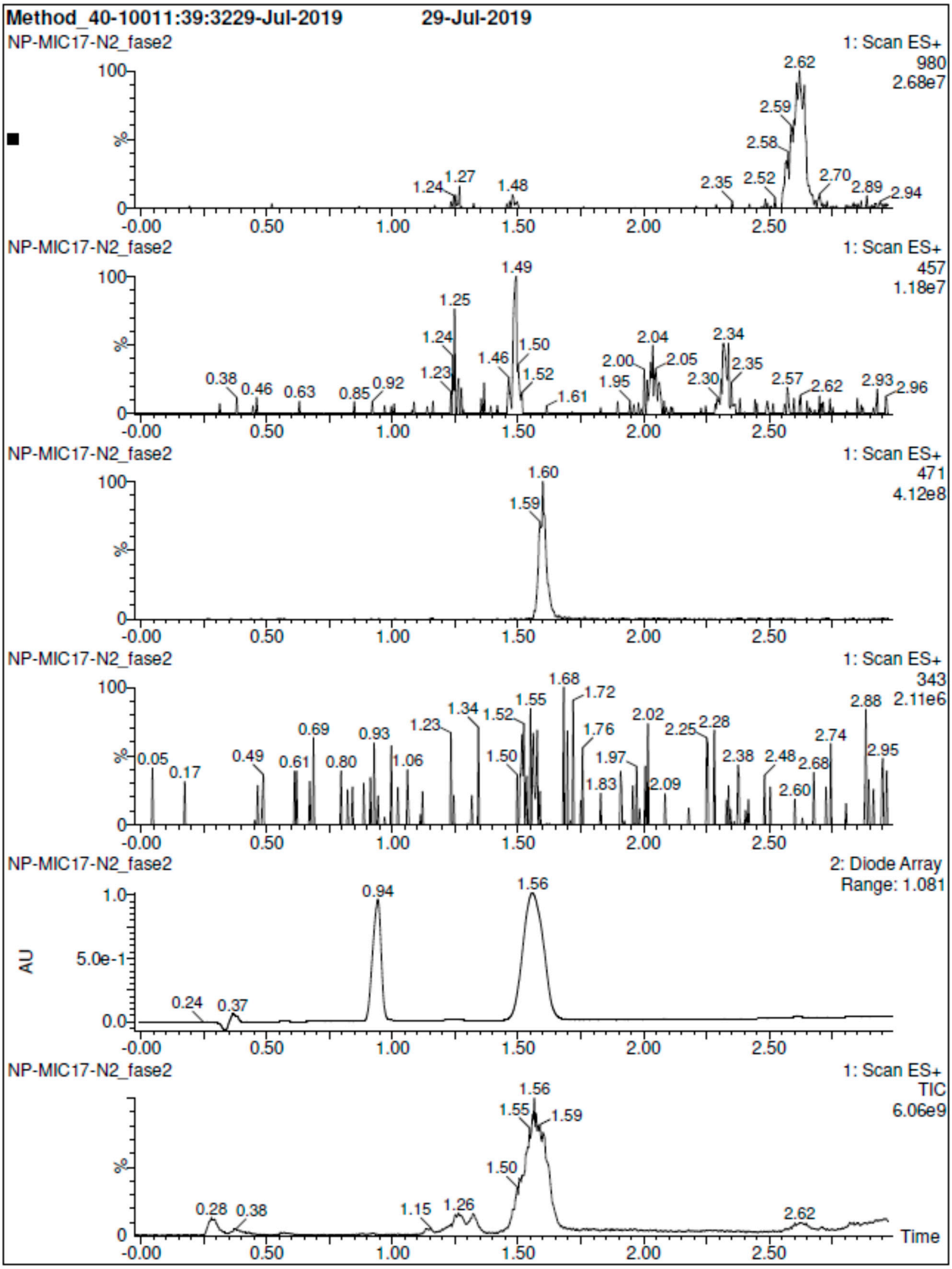

Figure S14. MS analysis / compound searching for **3-Be-mono** (MW = 979, first lane), betulinic acid (MW = 456, second lane), betulinic acid methyl ester (MW = 470, third lane) and trehalose (MW = 342 Da, fourth lane) in the lipophilic methanol-chloroform phase from the treatment of HeLa cells with **mono-Be-NA3/MIC-17**.
